# Comparison of outcomes of an 18-gauge vs 16-gauge ultrasound-guided percutaneous renal biopsy: a systematic review and meta-analysis

**DOI:** 10.1080/0886022X.2023.2257806

**Published:** 2023-09-19

**Authors:** Tingting Zhan, Ali Lou

**Affiliations:** Department of Ultrasound, The Second People’s Hospital of Lishui, Lishui, Zhejiang, China

**Keywords:** Meta-analysis, renal biopsy, ultrasonography

## Abstract

**Background:** The needle size used in ultrasound-guided percutaneous renal biopsy significantly influences the efficacy and safety of the procedure. The aim of this study is to perform a comparative analysis of 16-gauge and 18-gauge needles for ultrasound-guided percutaneous renal biopsy.

**Methods:** This systematic review and meta-analysis included randomized controlled trials and observational studies that compared the outcomes of using 18-gauge and 16-gauge needles for ultrasound-guided percutaneous renal biopsy. The efficacy parameters included a mean number of glomeruli obtained and the number of passes, while the safety parameters focused on the rate of complications. We searched multiple databases, assessed the risk of bias, and conducted statistical analyses using appropriate models.

**Results:** Fifteen studies were included. Compared to the 18-gauge needle, the use of 16-gauge needle for the biopsy was associated with the significantly higher mean number of glomeruli obtained (pooled SMD 0.61, 95%CI: 0.32 to 0.89; *p* < 0.001) and fewer required passes (pooled SMD −0.57, 95%CI: −0.97 to −0.18; *p* = 0.004). No significant difference was observed in the individual safety parameters, including pain, hematuria, need for blood transfusion, major, and minor complications. However, the use of 16-gauge needle was associated with higher odds of total complications (pooled OR 1.57, 95%CI: 1.16 to 2.13; *p* = 0.004).

**Conclusion:** While the 16-gauge needle for ultrasound-guided percutaneous renal biopsy offers improved efficacy in terms of a higher mean number of glomeruli and fewer required passes, it is associated with higher total complications. A judicious needle size selection that would consider patient-specific factors and risk-benefit ratio, is crucial for optimizing patient outcomes.

## Introduction

Percutaneous renal biopsy (PRB) remains an essential diagnostic and prognostic tool in the management of various renal diseases [1]. First introduced in the 1950s, PRB has undergone significant modifications and improvements. Recent advances in the imaging guidance and the use of ultrasound significantly enhanced the safety and efficacy of the procedure [2]. The application of ultrasound guidance for PRB has enabled real-time visualization, ensuring accurate needle placement, reducing risk of complications, and making it the method of choice in most medical facilities worldwide [3].

One aspect of PRB that has been a subject of ongoing debate is the selection of the biopsy needle gauge. The most used gauges are the 16-gauge and 18-gauge needles [4]. The gauge of a needle is a measure of its diameter: the larger the gauge number, the smaller the needle diameter. Therefore, a 18-gauge needle is smaller in diameter than a 16-gauge one [5]. The choice of needle gauge can influence several outcomes of PRB, including quality and adequacy of the biopsy specimen, rate of complications, as well as pain and discomfort of the patient [4].

Adequate tissue sampling is critical for the accurate diagnosis and management of renal disease [6]. Smaller gauge needles may yield less tissue, potentially affecting the diagnostic yield [7]. Conversely, larger gauge needles might provide a better tissue sample but could potentially result in a higher complication rate. Complications associated with PRB, although infrequent, may range from minor issues such as pain and hematuria to more serious ones, like perinephric hematoma, arteriovenous fistula, or, in extreme cases, life-threatening hemorrhage [8].

Although many studies [4, 7,8] have compared the 16-gauge and 18-gauge needles, there remains a lack of consensus on the optimal needle gauge for PRB. Given the significant implications of this decision for patient care, it is imperative to critically analyze the current evidence on this topic. This study aims to perform a systematic review and meta-analysis comparing the outcomes of 18-gauge versus 16-gauge ultrasound-guided PRB. Our results may provide more definitive guidance for clinicians on the choice of needle gauge in PRB, contributing to safer, more effective care for patients undergoing this procedure.

The findings of this study will be instrumental in standardizing PRB practices, leading to more accurate diagnosis and treatment of renal diseases, ultimately resulting in better patient outcomes. Moreover, they could inform guidelines on PRB and stimulate further research into refining this critical procedure.

## Methods

### Inclusion criteria

#### Meta-analysis registration

PROSPERO, https://www.crd.york.ac.uk/prospero/#searchadvanced, CRD42023433943

#### Study design

Eligible randomized controlled trials (RCT) either in parallel or in cluster form, observational studies (prospective and retrospective) were considered. We incorporated full-text studies that met the eligibility criteria, while case reports/series and unpublished grey literature were excluded from the analysis.

#### Study participants

Studies done in patients undergoing ultrasound-guided percutaneous renal biopsy were included.

#### Intervention and comparator groups

Studies, directly comparing the outcomes of 18-gauge vs 16-gauge needle for performing the ultrasound guided percutaneous renal biopsy were considered.

#### Outcomes

Efficacy parameters, such as: number of glomeruli obtained; number of passes.

Safety parameters in terms of the complications, such as pain, hematuria, need for blood transfusion, major complications, minor complications, and total number of complications.

##### Major complications

The presence of any of the following complications: massive bleeding requiring red blood cells (RBCs) transfusion, the requirement of angiographic embolization to control the bleeding, nephrectomy or death.

##### Minor complications

Renal hematoma following renal biopsy, pain and macroscopic hematuria, arteriovenous fistula and intravesical coagulation.

##### Total complications

Sum of patients having either major or minor complications.

### Search strategy

The search was conducted in multiple databases, including PubMed, SCOPUS, Web of Science, CINAHL, Cochrane Library, and trial registries. Our search strategy incorporated medical subject headings (MeSH) and free-text terms. We employed appropriate Boolean operators (‘AND,’ ‘OR,’ and ‘NOT’) to combine predefined search terms. The search period was from January 1964 (or inception of database, whichever is earlier) to May 2023, without any language restrictions (Supplementary File 1).

### Study selection

Two independent researchers conducted the initial stage of the study selection process by examining the titles, keywords, and abstracts. Both investigators obtained full-text studies and narrowed them down for the second phase of screening according to the eligibility criteria. In the second step, the researchers evaluated the retrieved full texts. Studies that met the eligibility criteria were ultimately included for further analysis. The ‘Preferred Reporting Items for Systematic Reviews and Meta-Analyses (PRISMA) checklist 2020’ was employed to report this review [9].

### Data extraction

Both researchers participated in the manual data extraction procedure, utilizing a predefined semi-structured data collection form.

### Risk of bias assessment

Two researchers undertook to assess the quality of the included studies using RoB 2 (‘Revised Cochrane risk-of-bias tool for randomized trials’) [10]. The RoB 2 tool encompasses five domains: ‘randomization process, deviations from intended interventions, missing outcome data, measurement of the outcome, and selection of the reported result’. Based on the responses, each study was categorized as having low, some concerns, or high risk of bias. The Newcastle Ottawa (NO) scale was used for assessing observational studies [11]. The NO scale encompasses selection, comparability, and outcome domains. Based on the responses, each study was categorized as having good/poor quality.

### Statistical analysis

The overall treatment effect was evaluated by calculating the combined values of weighted mean differences (WMD) or standardized mean differences (SMD) for the continuous outcomes. The random-effects model was used to pool the results that were then presented as forest plots, with 95% confidence intervals (Cis) for individual study estimates and pooled effect sizes. For the binary outcomes, the number of events and participants in each group was entered and analyzed. The pooled estimate was reported as an odds ratio with 95% CI. Heterogeneity was assessed using the I^2^ statistic, Chi-squared test, and visual inspection of the forest plot [12]. STATA version 14.2 was used for the analysis. Funnel plot and Egger’s test were done for outcomes with at least 10 studies to identify the possibility of publication bias.

## Results

### Search results

**Figure 1.**
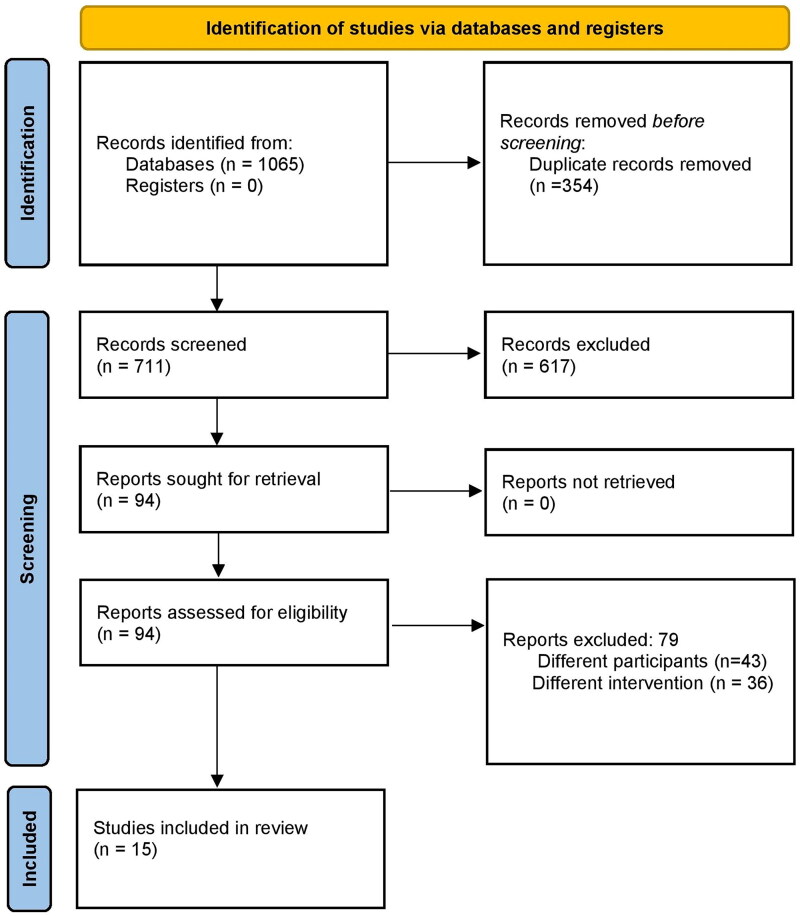
PRISMA Flow diagram for study selection.

Primary screening identified 1065 citations. Following duplicates removal, 94 full-text articles were retrieved. These studies underwent secondary screening and a total of 15 studies were included in the final analysis (Figure 1) [4, 7, 8, 13, 14, p. 2, 15–24].

### Characteristics of the included studies

More than half of the studies (8 out of 15) were retrospective. Sample sizes ranged from 50 to 3138. The mean age of the participants in the studies had wide variation, ranging from 8 to 55 years. Males were predominant in almost all the included studies when compared to females. Most included studies were conducted in Asian countries like China, India, and Japan followed by Western countries like United States of America, Sweden, and Canada. The definitions used for major, minor, and total complications are provided in Supplementary Table 1. Most studies (7 out of 15) had moderate risk of bias, two studies had a lower risk and the rest had a higher risk of bias **(**Table 1**)**.

**Table 1.** Characteristics of included studies (*N* = 15).

Author and Year	Study design	Country	Sample size	Mean age (In years)	Study participants	Renal biopsy details	Specialty operator	Male:Female	Outcomes reported	Risk of bias
Antunes et al. 2018 [8]	RCT	Brazil	18G-104 16G-134	Overall − 40.17	Participants with age > 12 years with indication of renal biopsy	Percutaneous technique with real-time ultrasound using Ultra-sound Xario XG and an automated device for automatic spring-loaded biopsy	NR	123:115	Mean number of glomeruli, major, minor & total complications	Some concerns
Arora et al. 2012 [13]	Prospective	India	18G-25 16G-25	NR	Patients who underwent renal biopsies from April 2007 until May 2008	NR	NR	NR	Mean number of glomeruli, pain, hematuria, major, minor & total complications	Low risk
Fatthy et al. 2022 [14]	Retrospective	Egypt	18G-451 16G-454	Overall − 45 years	Patients who had undergone renal biopsy between March 2013 and March 2018	Ultrasound guided percutaneous renal biopsy	Nephrologist	727:470	Major complications, hematuria	Moderate risk
Gupta et al. 2015 [15]	Prospective	UK	18G-39 16G-239	Overall − 10 years	Pediatric renal biopsies undertaken at participating centers between 1 January 2012 and 30 June 2012	NR	Nephrologist, radiologist or combination of nephrologist and radiologist	NR	Mean number of passes, major complications	High risk
Mai et al. 2013 [16]	Retrospective	Australia	18G-181 16G-753	18G-53 16G-51	Native-kidney percutaneous renal biopsy in south western Sydney between 2001 and 2010	All biopsies were performed under ultrasound guidance with Bard Magnum automatic biopsy devices	Nephrologist for 16 G needles Interventional Radiologist for 18 G needles	498:436	Mean number of glomeruli, hematuria, need for blood transfusion, major and total complications	Moderate
Nicholson et al. 2000 [17]	RCT	UK	18G-34 16G-33	18G-44 16G-40	Renal allograft recipients undergoing transplant biopsy	All biopsies were performed using real-time ultrasound guidance	Transplant surgeons	44:23	Mean number of glomeruli, pain, hematuria, need for blood transfusion	Some concerns
Nissen et al. 2022 [18]	Retrospective	USA	18G-185 16G-40	NR	All native renal biopsies from January 1, 2005 to December 31, 2020	All the samples were taken as per Ad Hoc Committee on Renal Biopsy Guidelines of the Renal Pathology Society Practice guidelines	Nephrologist or radiologist	NR	Mean number of glomeruli	High
Peters et al. 2016 [7]	Prospective & Retrospective	Sweden	18G = 264 16G = 230	18G = 50 16G = 54	Patients undergoing percutaneous renal biopsy	All biopsies were performed using real-time ultrasound guidance & automated spring-loaded device	Radiologists or nephrologists	320:174	Mean number of glomeruli, number of passes, major, minor & total complications	Moderate
Roth et al. 2013 [19]	Retrospective	USA	18G = 67 16G = 82	Overall − 55.2	Needle core biopsies of the renal cortex were obtained from a non-neoplastic area of kidney in nephrectomy	Three cores by each needle gauge were obtained from the same area of the renal cortex after the kidney was dissected by a frontal section.	Nephrologists and interventional radiologists	NR	Mean number of glomeruli, hematuria, need for blood transfusion	High
Sawicka et al. 2019 [20]	Retrospective	Canada	18G = 160 16G = 82	Overall − 49	Image-guided renal biopsies performed between 2012-2016	All biopsies were performed using real-time ultrasound guidance & automated spring-loaded device	Nephrologists and interventional radiologists	135:107	Mean number of glomeruli, total complications	Moderate
Sinha et al. 2016 [21]	Prospective	India	18G = 28 16G = 71	Overall − 8	Consecutive pediatric renal biopsies	All renal biopsies were performed under real‑time USG guidance	Pediatric nephrologist	NR	Mean number of glomeruli, hematuria, need for blood transfusion	Moderate
Sousanieh et al. 2020 [4]	Prospective	USA	18G = 131 16G = 892	18G = 51 16G = 53	Adult (≥15 years) patients from January 2002 to December 2019	The biopsy specimens were collected by a pathology lab technician and viewed under dissecting microscope	Nephrologist	541:482	Mean number of glomeruli, total complications, need for blood transfusion	Low
Tsuchida et al. 1997 [22]	Retrospective	Japan	18G = 19 16G = 28	18G = 9.5 16G = 8.1	Children, aged 2-15 years	Orthogonal ultrasound guided renal biopsy	NR	NR	Mean number of glomeruli	High
Xie et al. 2020 [23]	Retrospective	China	18G = 72 16G = 198	18G = 41 16G = 40	Patients aged 9-85 years undergoing percutaneous renal biopsy	The Pajunk Delta Cut Biopsy System was applied with the use of 16 G and 18 G biopsy needles	Nephrologist	152:118	Mean number of glomeruli, major, minor & total complications, pain, hematuria	High
Xu et al. 2022 [24]	Retrospective	China	18G = 2526 16G = 612	18G = 41.98 16G = 40.44	Patients who underwent percutaneous renal biopsy between January 2015 and December 2019	Biopsy was performed by a nephrologist, with an ultrasonologist in attendance to assist in real-time ultrasound guidance	Nephrologist with ultrasonographist	1431:1707	Mean number of glomeruli, pain, hematuria, need for blood transfusion	High

18G-18 Gauge; 16 G-16 Gauge; NR – Not reported; RCT – Randomized controlled trial; UK-United Kingdom; USA – United States of America.

### Efficacy parameters

#### Mean number of obtained glomeruli

**Figure 2.**
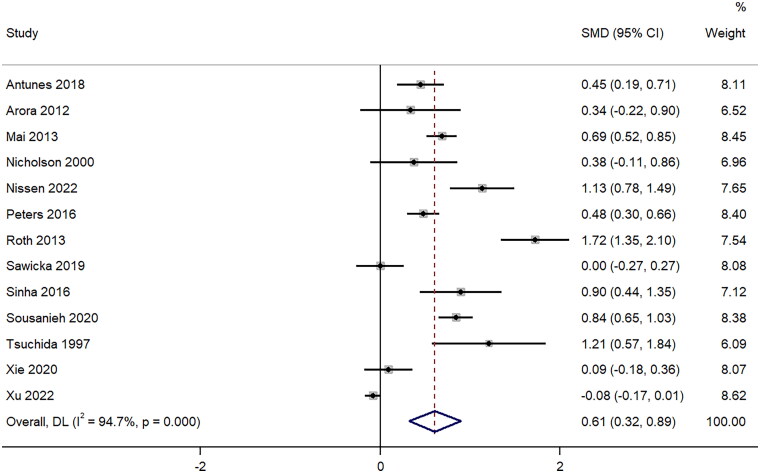
Forest Plot of Standardized Mean difference in number of glomeruli retrieved with 16-Gauge vs. 18-Gauge biopsy needles.

A total of 13 studies provided information on the difference in the mean number of obtained glomeruli between ultrasound-guided PRB using 18-gauge and the 16-gauge needles. The pooled SMD was 0.61 (95%CI: 0.32 to 0.89; I^2^=94.7%), indicating that there is a substantial increase in the mean number of glomeruli that were retrieved by the 16-gauge needle-guided biopsy when compared to the 18-gauge needle one (*p* < 0.001) (Figure 2). The funnel plot was asymmetrical with significant Egger’s test (*p* = 0.02), indicating the possibility of publication bias (Supplementary Figure 1). Sensitivity analysis (Supplementary Figure 2) showed a lack of single-study effect on the final pooled effect size.

#### Mean number of passes

**Figure 3.**
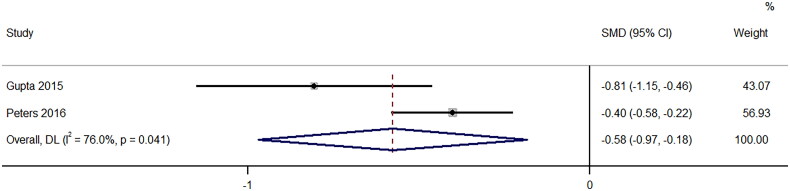
Forest Plot of Standardized Mean difference in number of passes required with 16-Gauge vs. 18-Gauge biopsy needles.

A total of 13 studies provided information on the difference in the mean number of passes between ultrasound-guided PRB with 18-gauge and 16-gauge needles. The pooled SMD was −0.57 (95%CI: −0.97 to −0.18; I^2^=76%), signifying a substantial decrease in the mean number of passes when 16-gauge needle was used compared to 18-gauge (*p* = 0.004) (Figure 3).

### Safety parameters

#### Pain

**Figure 4.**
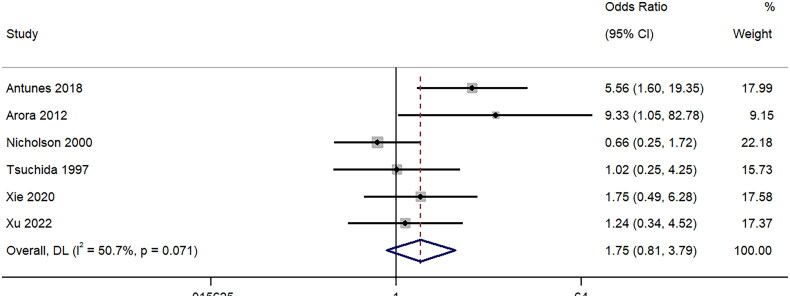
Forest Plot of Odds Ratio for Pain experienced during biopsy with 16-Gauge vs. 18-Gauge needles.

Six studies provided information on the differences in pain in patients who underwent ultrasound-guided PRB with the 18-gauge and the 16-gauge needles. The pooled OR was 1.75 (95%CI: 0.81 to 3.79; I^2^=50.7%), signifying no difference between the two groups (*p* = 0.16) (Figure 4). Sensitivity analysis (Supplementary Figure 3) showed a lack of single-study effect on the final pooled effect size.

### Hematuria

**Figure 5.**
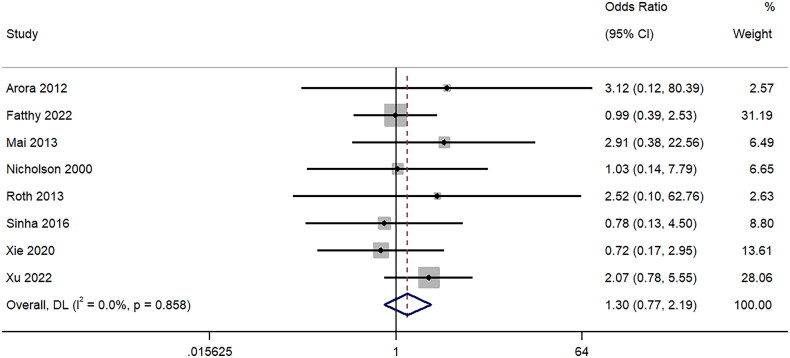
Forest Plot of Odds Ratio for Hematuria following biopsy with 16-Gauge vs. 18-Gauge needles.

Eight studies provided information on the difference in hematuria between 18-gauge and 16-gauge ultrasound-guided percutaneous renal biopsy. The pooled OR of 1.30 (95%CI: 0.77 to 2.19; I^2^=0%) showed no difference between the two groups (*p* = 0.33) (Figure 5). Sensitivity analysis (Supplementary Figure 4) did not detect single study effect on the final pooled effect size.

### Need for blood transfusion

**Figure 6.**
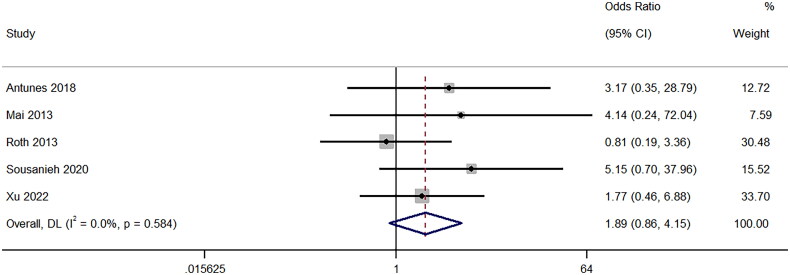
Forest Plot of Odds Ratio for Need for blood transfusion following biopsy with 16-Gauge vs. 18-Gauge needles.

A total of five studies provided information on the different need for blood transfusion between 18-gauge and 16-gauge ultrasound-guided PRB groups of patients. The pooled OR was 1.89 (95%CI: 0.86 to 4.15; I^2^ = 0%), indicating comparable results for both groups (*p* = 0.11) (Figure 6). Sensitivity analysis (Supplementary Figure 5) showed that the exclusion of [19] was able to widen the upper interval of pooled effect size. However, there was no impact on the direction of association.

### Major complications

**Figure 7.**
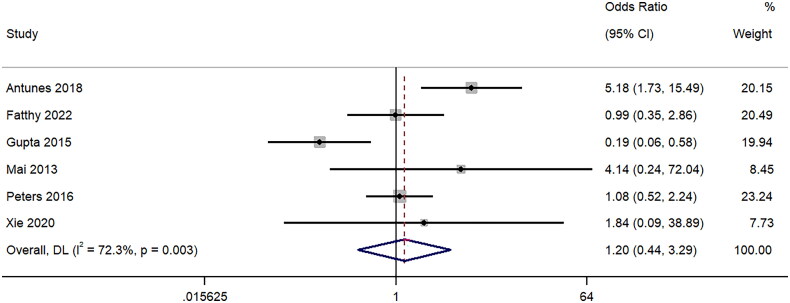
Forest Plot of Odds Ratio for Major complications Post-Biopsy with 16-Gauge vs. 18-Gauge needles.

The difference in major complications associated with the biopsy using 18-gauge and 16-gauge needles was reported in six studies. The pooled OR was 1.20 (95%CI: 0.44 to 3.29; I^2^ = 72.3%), signifying no difference between the two groups (*p* = 0.72) (Figure 7). Sensitivity analysis (Supplementary Figure 6) demonstrated lack of single study effect on the final pooled effect size.

### Minor complications

**Figure 8.**
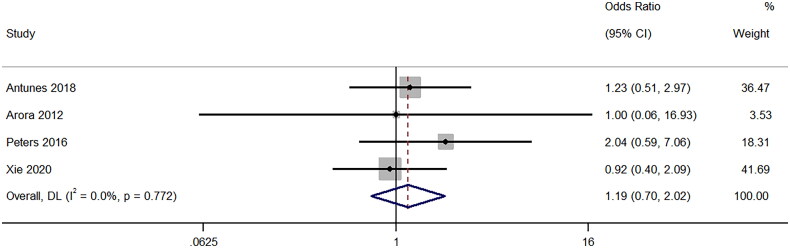
Forest Plot of Odds Ratio for Minor complications Post-Biopsy with 16-Gauge vs. 18-Gauge needles.

Four studies compared the rates of minor complications and showed that there was no difference between the groups, with the pooled OR of 1.19 (95%CI: 0.70 to 2.02; I^2^ = 0%), (*p* = 0.53) (Figure 8). Sensitivity analysis (Supplementary Figure 7) also showed a lack of single-study effect on the final pooled effect size.

### Total complications

**Figure 9.**
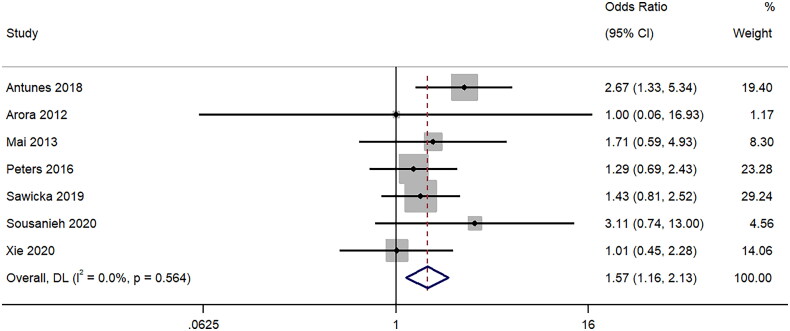
Forest Plot of Odds Ratio for Total complications Post-Biopsy with 16-Gauge vs. 18-Gauge needles.

Seven studies provided information on the difference in the rate of total complications associated with the biopsy using 18-gauge and 16-gauge needles. The pooled OR was 1.57 (95%CI: 1.16 to 2.13; I^2^ = 0%). This shows that there is a higher rate of total complications associated with 16-gauge compared to 18-gauge ultrasound-guided percutaneous renal biopsy (*p* = 0.004) (Figure 9). Sensitivity analysis (Supplementary Figure 8) demonstrated that exclusion of [8] changes the direction of association from significant to non-significant pooled effect size for total complications.

## Discussion

This systematic review and meta-analysis aimed to explore the comparative effectiveness and safety of using 16-gauge needles versus 18-gauge needles in ultrasound-guided percutaneous renal biopsies. The main findings of our study indicate that using a 16-gauge needle yielded significantly more glomeruli and required fewer passes, thus potentially increasing the diagnostic yield, and reducing the duration of the procedure. Nevertheless, our analysis also revealed a potential increase in the incidence of total complications when using 16-gauge needles, a finding that warrants further consideration.

The use of 16-gauge needle was associated with an increased mean number of obtained glomeruli, which is consistent with previous literature included in the current review [8, 13, 16, 18–20, 22, 24]. Theoretically, the larger lumen of the 16-gauge needle should allow for more tissue to be harvested during each pass, thus increasing the potential for obtaining more glomeruli. However, contrary to our study findings, [24] found no significant difference in the number of glomeruli obtained between 16- and 18-gauge needles. This discrepancy might be attributed to sample size or technique variations across the studies. From a clinical standpoint, these efficacy parameters have critical implications. An increased number of obtained glomeruli potentially enhances the diagnostic accuracy of renal biopsies. This is vital in several pathological conditions, where a greater tissue yield may lead to more precise histopathological characterization, aiding in a more definitive diagnosis and targeted therapeutic approach.

Simultaneously, we observed fewer passes required with the 16-gauge needle. Our results align with the previous studies [15,7] that reported a reduced number of passes needed when 16-gauge needles were used. A reduced number of passes not only shortens the time of the procedure but may also enhance patient comfort and compliance [25]. This could be particularly beneficial in patients with anxiety or those with poor tolerance to invasive procedures. Furthermore, fewer passes might decrease the likelihood of needle-track seeding, especially in the context of malignant renal conditions. The predominant indication across the included studies was diffuse renal diseases. However, it’s important to acknowledge that renal biopsies might also be indicated for focal renal lesions, including potential neoplasms. The needle-track seeding, especially with larger gauge needles, is a point of concern when biopsy for potentially malignant tumors. Such seeding might have implications on tumor spread and overall prognosis. Understanding the exact indication for biopsy and the nature of the lesion – diffuse versus focal, benign versus potentially malignant – is crucial in determining the optimal gauge of the needle. It can influence the potential risks associated with the procedure and guide the clinician in ensuring that the benefits of the biopsy outweigh its potential downsides.

However, the potential benefits of the 16-gauge needle must be balanced against its potential downsides. In terms of safety parameters, our findings highlighted an interesting nuance: the risk of individual complications such as pain, hematuria, and need for blood transfusion, and both major and minor complications did not significantly differ between the two needle sizes. This contradicts the theoretical assumption that larger needles could lead to more damage and cause more complications [26]. However, when we assessed the rate of total complications, we found higher odds associated with the 16-gauge needle. One could argue that the cumulative risk from various minor complications might result in a significantly higher total complication rate for the 16-gauge needle, suggesting a tradeoff between higher efficacy (increased glomeruli yield and fewer passes) and safety. Understanding this nuanced relationship between efficacy and safety is vital in clinical decision-making, particularly in patients with preexisting conditions, such as coagulation disorders or renal inflammation that might predispose them to higher risk of complications.

In our systematic review and meta-analysis, a pertinent observation was the slightly increased total complications associated with the 16-gauge needles, even though major and minor complications were statistically similar between the two needle gauges. Diving deeper into this topic is crucial for a holistic understanding of the findings.

The nature of complications arising from renal biopsies can be multifaceted. While the core analysis indicated an uptick in total complications with 16-gauge needles, the sensitivity analysis offered a different perspective. Notably, upon the exclusion of the study by [8], the direction of the association between needle size and total complications pivoted from being significant to non-significant. This suggests that the results may hinge considerably on individual studies, emphasizing the need for cautious interpretation and the potential value of further primary research on the topic.

Antunes et al.’s study differentiated itself by noting a statistically significant difference in complications primarily due to hemodynamic stability and pain. Hemodynamic instability, characterized by transient alterations in blood pressure or heart rate, can be a reflection of pain or discomfort during the procedure. The larger lumen of the 16-gauge needle might be a contributing factor. It’s plausible that the broader puncture could induce more pain in some patients, leading to transient hemodynamic changes. This aspect, combined with the larger tissue sample that the 16-gauge needle secures, may also lead to a slightly increased risk of bleeding, further contributing to hemodynamic alterations.

Our results have direct clinical implications. Irrespective of our findings, it’s paramount to emphasize that the choice between using a 16-G or 18-G needle should always be individualized, considering each patient’s unique clinical circumstances and risk profile. The increased diagnostic yield with the 16-gauge needles may be preferred in cases where a definitive diagnosis is elusive and requires an extensive tissue sample. However, in patients with a higher risk of complications, an 18-gauge needle might be more appropriate to minimize the risk of total complications. The selection between the 16 G and 18 G needles, especially in non-randomized studies, may be influenced by various factors that warrant exploration.

One of the most pertinent considerations driving the choice of needle gauge, as reported by the included studies, is the perceived risk of complications, especially bleeding. The smaller diameter of the 18 G needle is often chosen with the anticipation of reduced tissue trauma, which in turn might translate to a lesser risk of hemorrhage, both immediate and delayed. This might particularly be the case in settings where patients are considered at a higher baseline risk of bleeding, either due to concurrent medication, underlying pathology, or coagulation profile anomalies. It’s worth noting that in many clinical contexts, the decision to opt for an 18-G needle is often a tradeoff between potentially compromised biopsy yield and perceived enhanced safety.

The main strength of our review is its rigorous methodological approach, comprehensive search strategies, strict adherence to PRISMA guidelines, and an in-depth risk of bias assessment. However, several limitations must be acknowledged. We report the presence of the risk of publication bias, as detected by the asymmetrical funnel plot and significant Egger’s test in the analysis of the mean number of glomeruli. This may overestimate the efficacy of the 16-gauge needles. Also, high heterogeneity in some of our analyses, despite using a random-effects model, suggests the presence of substantial variation across the included studies that cannot be explained solely by chance. Another notable limitation is the heterogeneity in measurement tools used across the analyzed studies. Given that varying pain-assessment scores and other outcome measures were employed, the comparisons and aggregations made may not fully capture the nuances of each individual study’s findings. This disparity in measurement methods can potentially introduce bias in our results and interpretations. The included studies in our meta-analysis comprised both native and transplant kidneys, and unfortunately, distinct outcome values for each group were not reported separately in the individual studies. This precluded our ability to perform a subgroup analysis based on this distinction. We recognize the potential impact of this limitation, especially in terms of generalizing our findings, and recommend future research that delves deeper into this aspect, allowing for a more stratified understanding of the implications of needle gauge choice in these different populations.

Future studies need to explore the potential correlation between the number of passes during the biopsy procedure and the subsequent complications. Intuitively, an increase in the number of passes might be associated with a higher risk of complications. Every additional pass implies repeated tissue trauma and potentially elevates the risk of hemorrhage, infection, and other associated adverse events. Furthermore, multiple passes might also heighten the patient’s discomfort and anxiety, both of which are crucial to consider from a holistic patient care perspective.

Future research should also focus on identifying patient subgroups that might particularly benefit from one needle size over the other. Long-term follow-up studies would be beneficial to monitor the incidence of late-onset complications and to further investigate the risk-benefit ratio. High-quality RCTs are necessary to compare the two interventions rigorously, taking into consideration patient comfort and satisfaction, cost-effectiveness, and operator experience and preference.

In conclusion, this systematic review and meta-analysis provide evidence that supports the use of 16-gauge needles in ultrasound-guided percutaneous renal biopsies for obtaining a greater mean number of glomeruli and reducing the number of passes. However, the potential increase in total complications should be cautiously considered during needle selection. More high-quality research is needed to further investigate these findings and develop clear-cut recommendations for clinical practice.

## Supplementary Material

Supplemental Material
