# Supplementary material for: Comparison of outcomes of an 18-gauge vs 16-gauge ultrasound-guided percutaneous renal biopsy: a systematic review and meta-analysis
Source: Ren Fail. 2023 Sep 19;45(2):2257806. doi: 10.1080/0886022X.2023.2257806 (PMC10512899; doi:10.1080/0886022X.2023.2257806)
Supplement: Supplemental Material [file IRNF_A_2257806_SM9040.zip › Supplementary Table 1.docx]

**Supplementary Table 1: Definitions of major, minor and total complications amongst the included studies**

| **Study** | **Major complications** | **Minor complications** | **Total complications** |
| --- | --- | --- | --- |
| Antunes 2018 | Disabling intense pain, hemodynamic instability with hemotransfusion and hemotransfusion with embolization | Arteriovenous fistula and intravesical coagulation | Sum of major and minor complications |
| Arora 2012 | NR | Pain and microscopic haematuria | Any complications |
| Fatthy 2022 | Need for blood transfusion or the need for surgical procedure/angiographic embolisation to stop the bleeding following the biopsy procedure | NA | NA |
| Gupta 2015 | Requirement for blood transfusion and/or surgical  exploration, delay in discharge or readmission for  observation | NA | NA |
| Mai 2013 | Patients with retroperitoneal haemorrhage and or require blood transfusion | NA | NR |
| Nicholson 2000 | NA | NA | NA |
| Nissen 2022 | NA | NA | NA |
| Peters 2016 | Bleeding requiring blood transfusion, acute renal obstruction, septicaemia | Gross hematuria, perinephric hematoma | Sum of major and minor complications |
| Roth 2013 | NA | NA | NA |
| Sawicka 2019 | NR | NR | Sum of major and minor complications |
| Sinha 2016 | NA | NA | NA |
| Sousanieh 2020 | NR | NR | Sum of major and minor complications |
| Tsuchida 1997 | NA | NA | NA |
| Xie 2020 | Bleeding requiring surgical intervention or  blood transfusion, a decline in blood pressure, a  decline in hemoglobin levels, severe infection | Lumbar or abdominal pain, hematuria, and perirenal hematoma | Sum of major and minor complications |
| Xu 2022 | NA | NA | NA |

NR – Not reported; NA – Not applicable
