# Supplementary material for: Comparison of outcomes of an 18-gauge vs 16-gauge ultrasound-guided percutaneous renal biopsy: a systematic review and meta-analysis
Source: Ren Fail. 2023 Sep 19;45(2):2257806. doi: 10.1080/0886022X.2023.2257806 (PMC10512899; doi:10.1080/0886022X.2023.2257806)
Supplement: Supplemental Material [file IRNF_A_2257806_SM9040.zip › Supplementary_Appendix.docx]

**Supplementary Appendix**:

**PubMed Search strategy**:

"18"[All Fields] AND ("gauge"[All Fields] OR "gauged"[All Fields] OR "gauges"[All Fields]

OR "gauging"[All Fields]) AND "16"[All Fields] AND ("gauge"[All Fields] OR

"gauged"[All Fields] OR "gauges"[All Fields] OR "gauging"[All Fields]) AND ("diagnostic imaging"[MeSH Subheading] OR ("diagnostic"[All Fields] AND "imaging"[All Fields]) OR

"diagnostic imaging"[All Fields] OR "ultrasound"[All Fields] OR "ultrasonography"[MeSH

Terms] OR "ultrasonography"[All Fields] OR "ultrasonics"[MeSH Terms] OR

"ultrasonics"[All Fields] OR "ultrasounds"[All Fields] OR "ultrasound s"[All Fields]) AND

("guide"[All Fields] OR "guided"[All Fields] OR "guides"[All Fields] OR "guiding"[All

Fields]) AND ("percutaneous"[All Fields] OR "percutaneously"[All Fields] OR

"percutanous"[All Fields]) AND ("renal"[All Fields] OR "renals"[All Fields]) AND

("biopsie"[All Fields] OR "biopsy"[MeSH Terms] OR "biopsy"[All Fields] OR

"biopsied"[All Fields] OR "biopsies"[All Fields] OR "biopsy s"[All Fields] OR

"biopsying"[All Fields] OR "biopsys"[All Fields] OR "pathology"[MeSH Subheading] OR

"pathology"[All Fields]) **Translations**

**gauge**: "gauge"[All Fields] OR "gauged"[All Fields] OR "gauges"[All Fields] OR

"gauging"[All Fields]

**gauge**: "gauge"[All Fields] OR "gauged"[All Fields] OR "gauges"[All Fields] OR

"gauging"[All Fields]

**ultrasound**: "diagnostic imaging"[Subheading] OR ("diagnostic"[All Fields] AND

"imaging"[All Fields]) OR "diagnostic imaging"[All Fields] OR "ultrasound"[All Fields] OR

"ultrasonography"[MeSH Terms] OR "ultrasonography"[All Fields] OR "ultrasonics"[MeSH Terms] OR "ultrasonics"[All Fields] OR "ultrasounds"[All Fields] OR "ultrasound's"[All Fields]

**guided**: "guide"[All Fields] OR "guided"[All Fields] OR "guides"[All Fields] OR

"guiding"[All Fields]

**percutaneous**: "percutaneous"[All Fields] OR "percutaneously"[All Fields] OR

"percutanous"[All Fields]

**renal**: "renal"[All Fields] OR "renals"[All Fields]

**biopsy**: "biopsie"[All Fields] OR "biopsy"[MeSH Terms] OR "biopsy"[All Fields] OR "biopsied"[All Fields] OR "biopsies"[All Fields] OR "biopsy's"[All Fields] OR

"biopsying"[All Fields] OR "biopsys"[All Fields] OR "pathology"[Subheading] OR "pathology"[All Fields]

**EMBASE:**

(('18':ab,ti OR '18 gauge':ab,ti OR '18 gauged':ab,ti OR '18 gauges':ab,ti OR '18 gauging':ab,ti) AND ('16':ab,ti OR '16 gauge':ab,ti OR '16 gauged':ab,ti OR '16 gauges':ab,ti OR '16 gauging':ab,ti)) AND ('diagnostic imaging':ab,ti OR 'diagnostic':ab,ti AND

'imaging':ab,ti OR 'ultrasound':ab,ti OR 'ultrasonography':ab,ti OR 'ultrasonics':ab,ti OR

'ultrasounds':ab,ti OR 'ultrasound s':ab,ti) AND ('guide':ab,ti OR 'guided':ab,ti OR

'guides':ab,ti OR 'guiding':ab,ti) AND ('percutaneous':ab,ti OR 'percutaneously':ab,ti OR

'percutanous':ab,ti) AND ('renal':ab,ti OR 'renals':ab,ti) AND ('biopsie':ab,ti OR 'biopsy':ab,ti

OR 'biopsied':ab,ti OR 'biopsies':ab,ti OR 'biopsy s':ab,ti OR 'biopsying':ab,ti OR

'biopsys':ab,ti OR 'pathology':ab,ti)
