# Supplementary material for: Comparison of outcomes of an 18-gauge vs 16-gauge ultrasound-guided percutaneous renal biopsy: a systematic review and meta-analysis
Source: Ren Fail. 2023 Sep 19;45(2):2257806. doi: 10.1080/0886022X.2023.2257806 (PMC10512899; doi:10.1080/0886022X.2023.2257806)
Supplement: Supplemental Material [file IRNF_A_2257806_SM9040.zip › Supplementary_Figure_Captions.docx]

Supplementary Figure 1: Funnel Plot for Publication Bias Evaluation for Studies Reporting Mean Number of Glomeruli

Supplementary Figure 2: Sensitivity Analysis for Studies Reporting Mean Number of Glomeruli

Supplementary Figure 3: Sensitivity Analysis for Studies Reporting Pain Post-Biopsy

Supplementary Figure 4: Sensitivity Analysis for Studies Reporting Haematuria Post-Biopsy

Supplementary Figure 5: Sensitivity Analysis for Studies Reporting Need for Blood Transfusion

Supplementary Figure 6: Sensitivity Analysis for Studies Reporting Major Complications

Supplementary Figure 7: Sensitivity Analysis for Studies Reporting Minor Complications

Supplementary Figure 8: Sensitivity Analysis for Studies Reporting Total Complications
