# Supplementary figures and images for: Comparison of outcomes of an 18-gauge vs 16-gauge ultrasound-guided percutaneous renal biopsy: a systematic review and meta-analysis
Source: Ren Fail. 2023 Sep 19;45(2):2257806. doi: 10.1080/0886022X.2023.2257806 (PMC10512899; doi:10.1080/0886022X.2023.2257806)

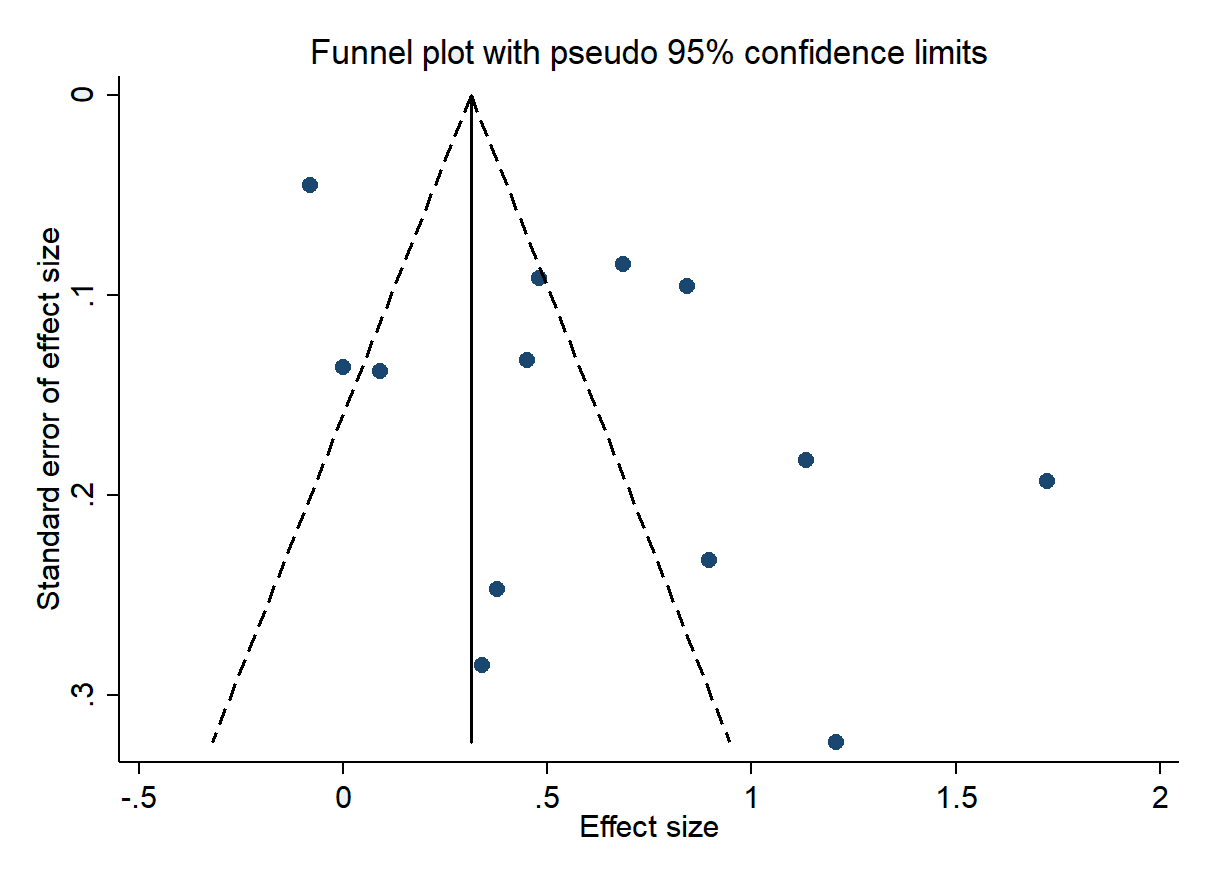

Supplement: Supplemental Material [file IRNF_A_2257806_SM9040.zip › Supplementary Figure 1.tif]

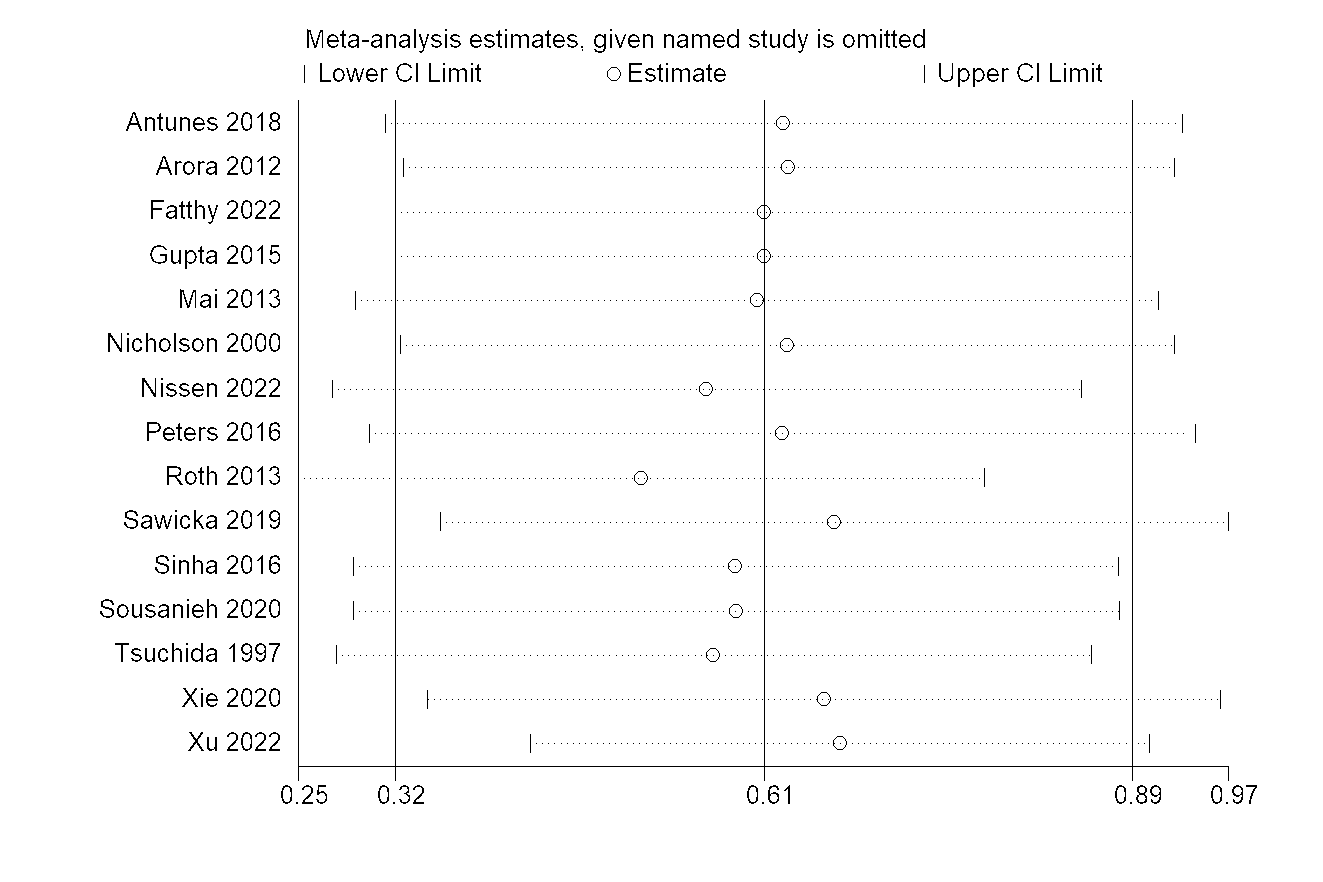

Supplement: Supplemental Material [file IRNF_A_2257806_SM9040.zip › Supplementary Figure 2.tif]

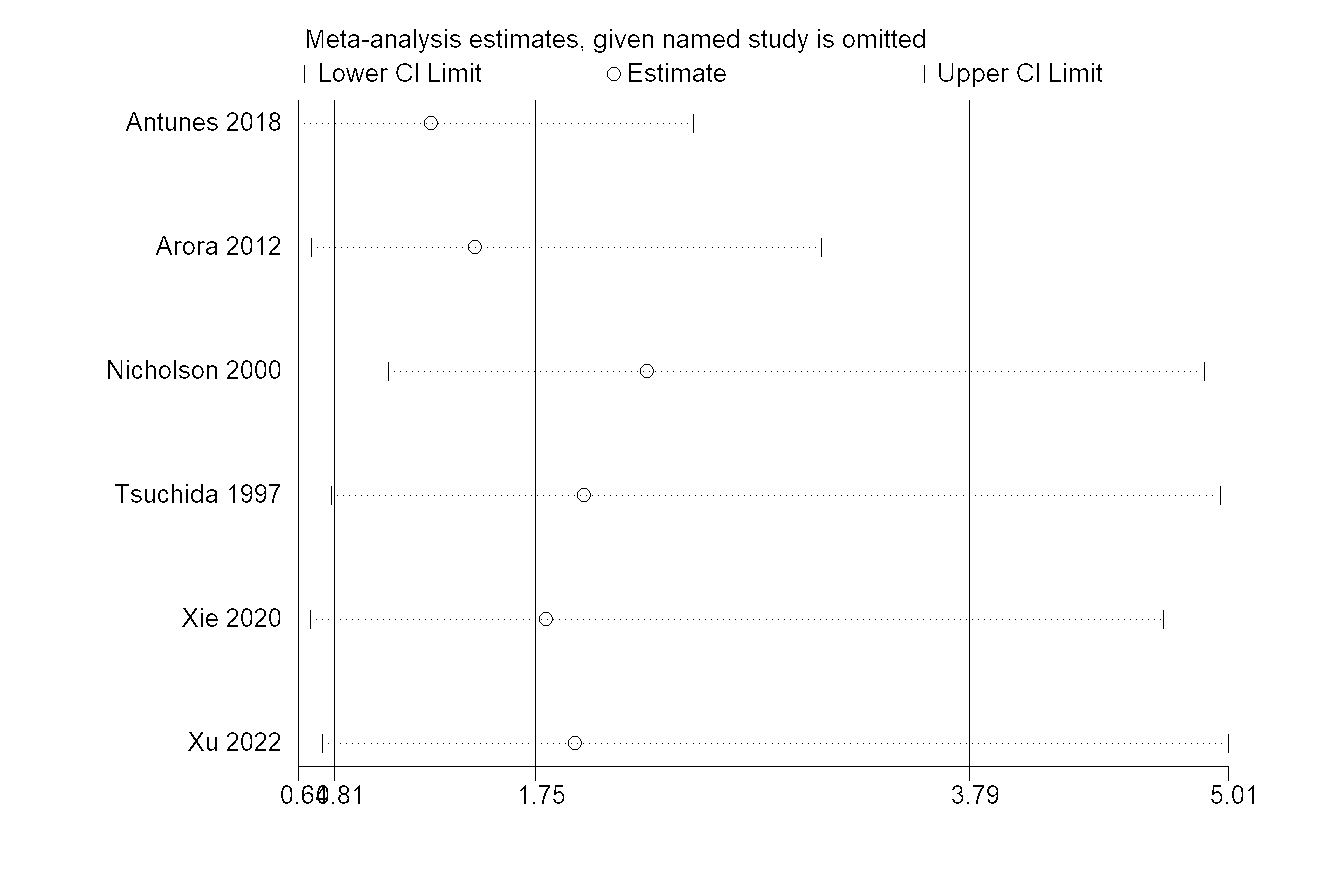

Supplement: Supplemental Material [file IRNF_A_2257806_SM9040.zip › Supplementary Figure 3.tif]

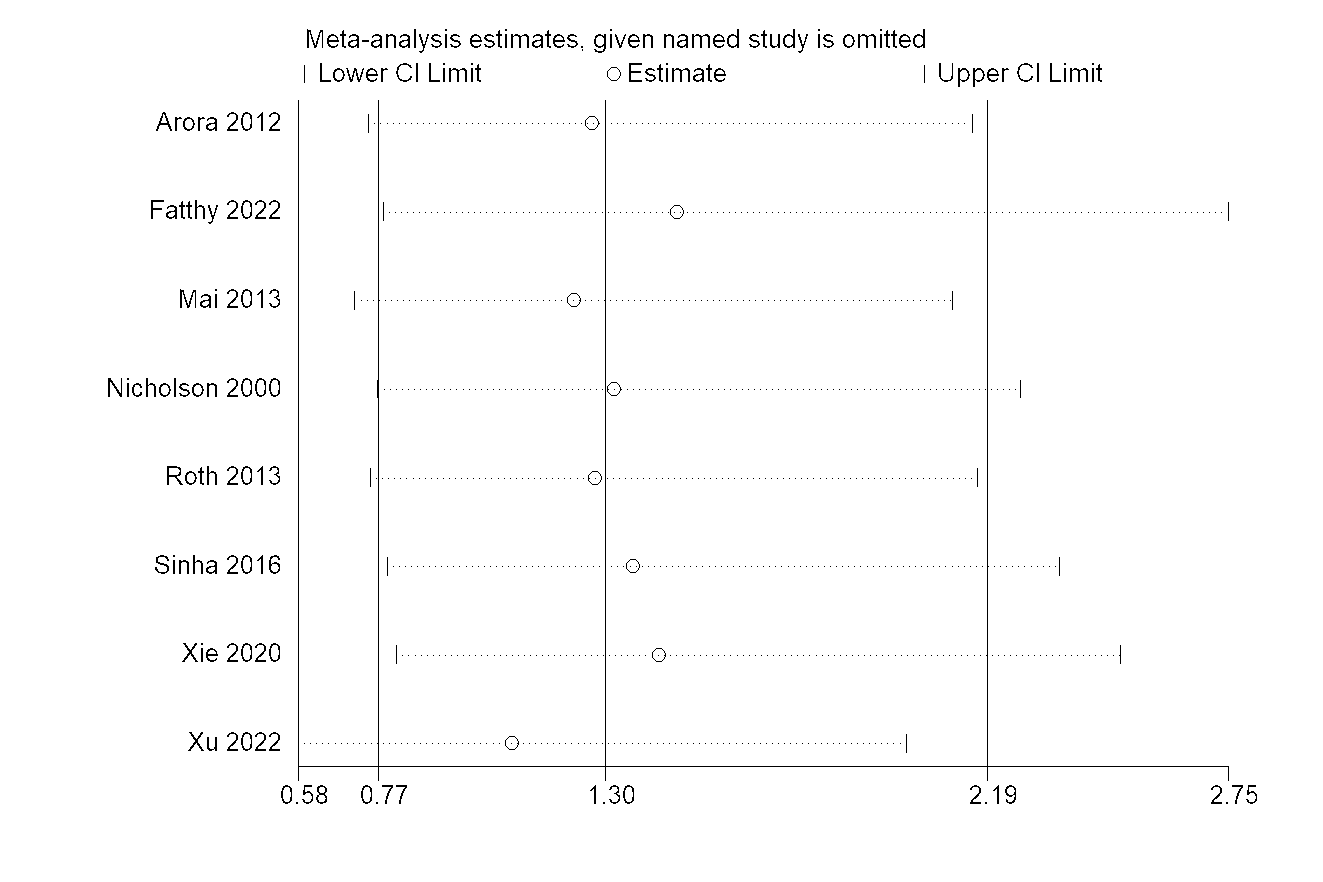

Supplement: Supplemental Material [file IRNF_A_2257806_SM9040.zip › Supplementary Figure 4.tif]

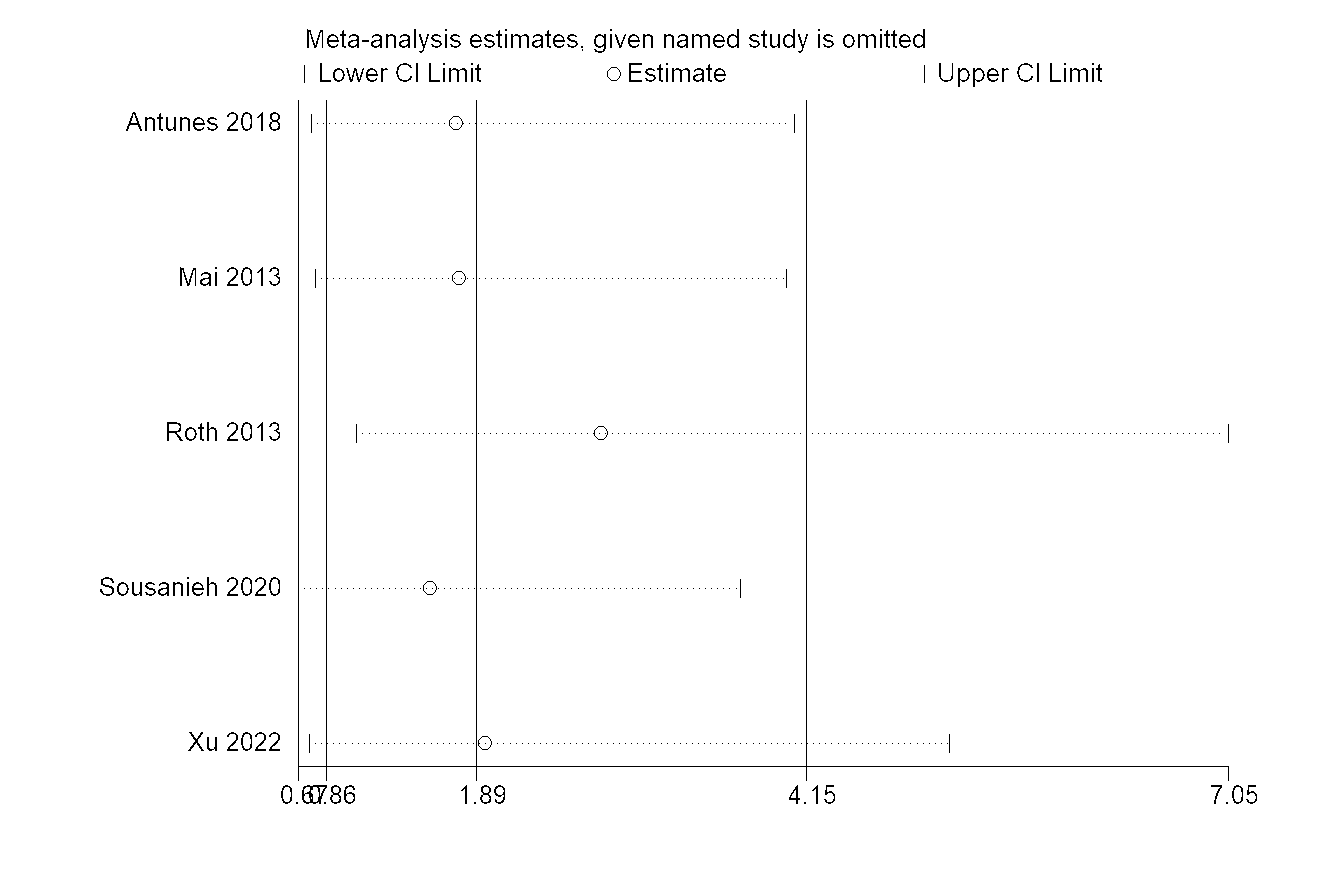

Supplement: Supplemental Material [file IRNF_A_2257806_SM9040.zip › Supplementary Figure 5.tif]

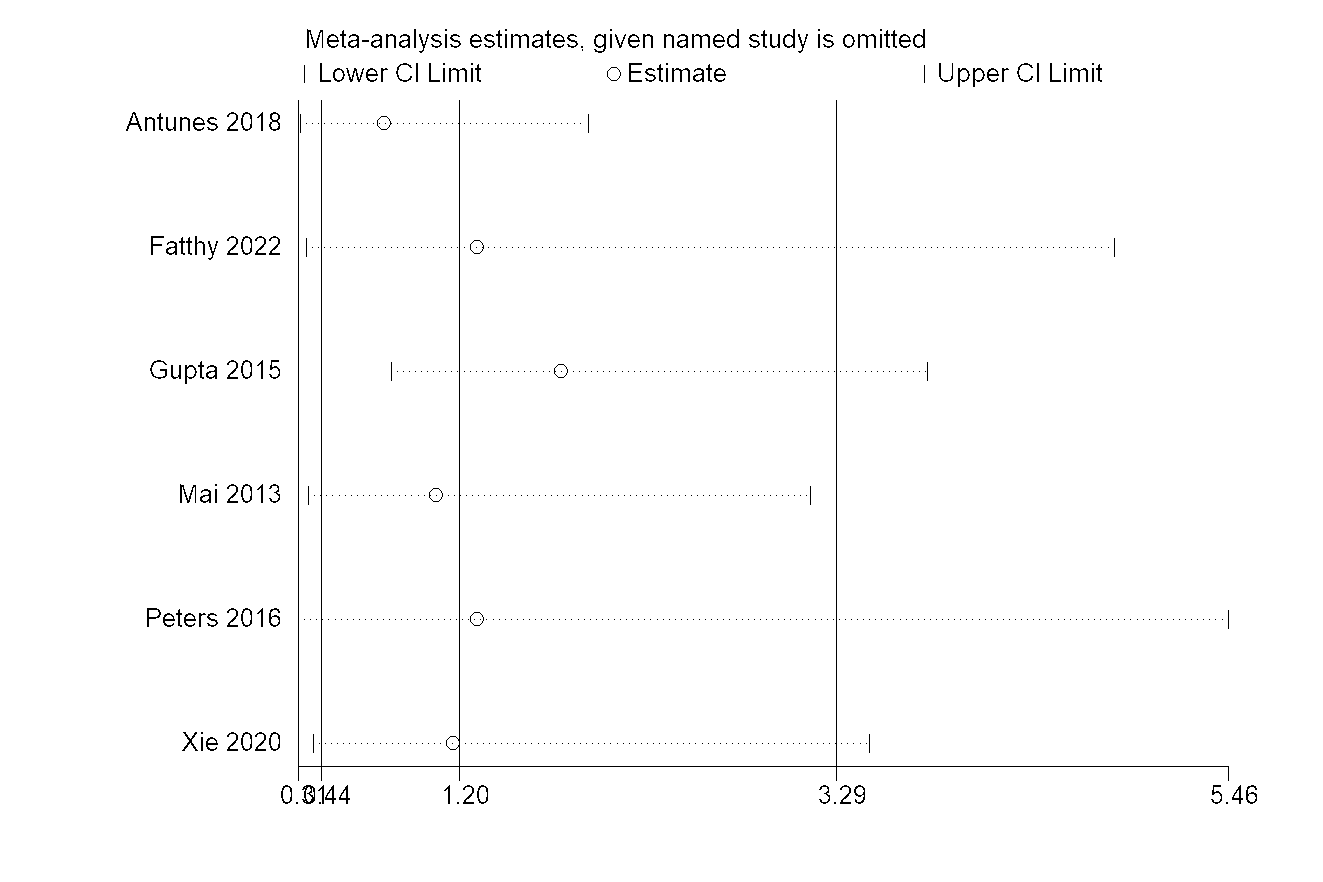

Supplement: Supplemental Material [file IRNF_A_2257806_SM9040.zip › Supplementary Figure 6.tif]

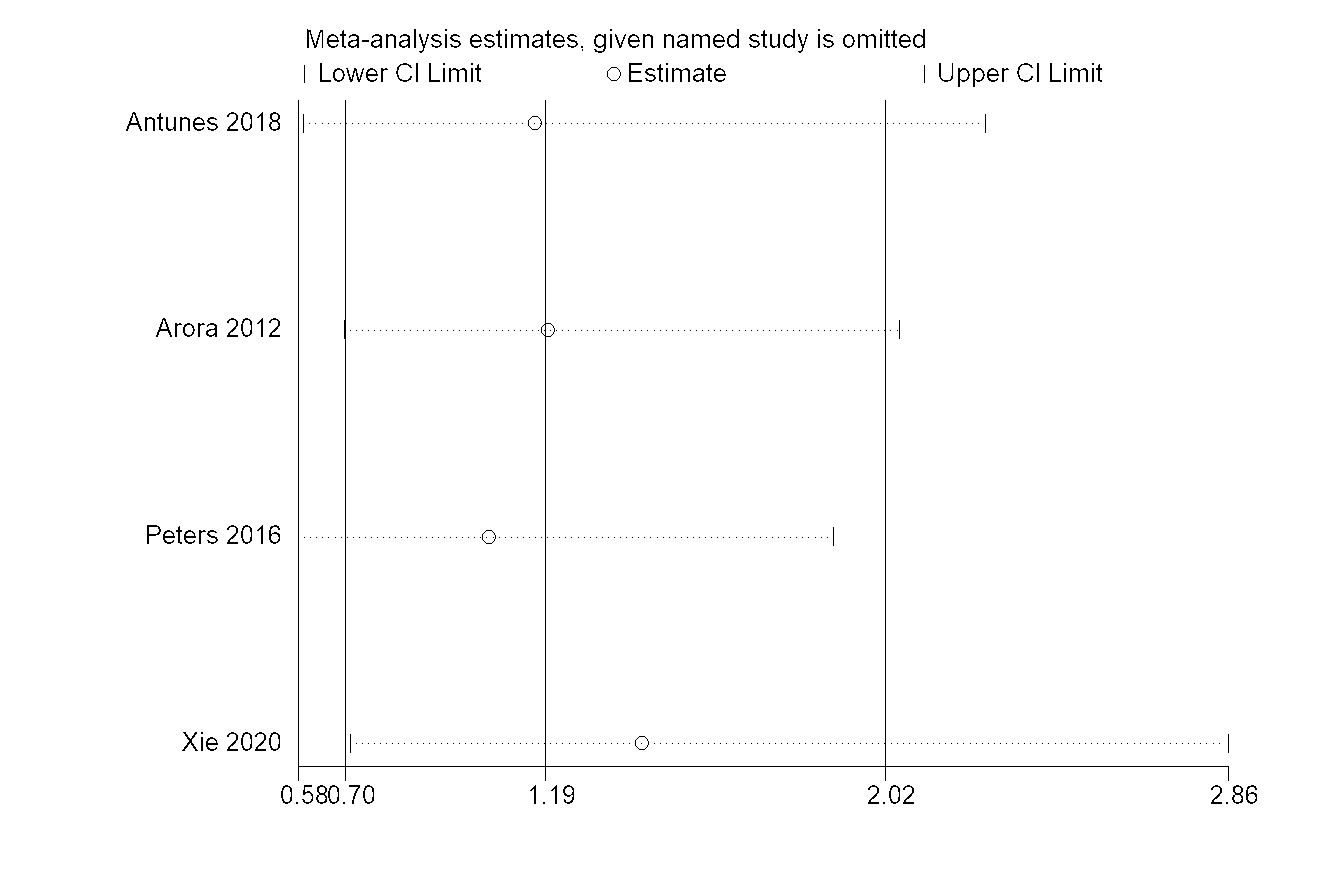

Supplement: Supplemental Material [file IRNF_A_2257806_SM9040.zip › Supplementary Figure 7.tif]

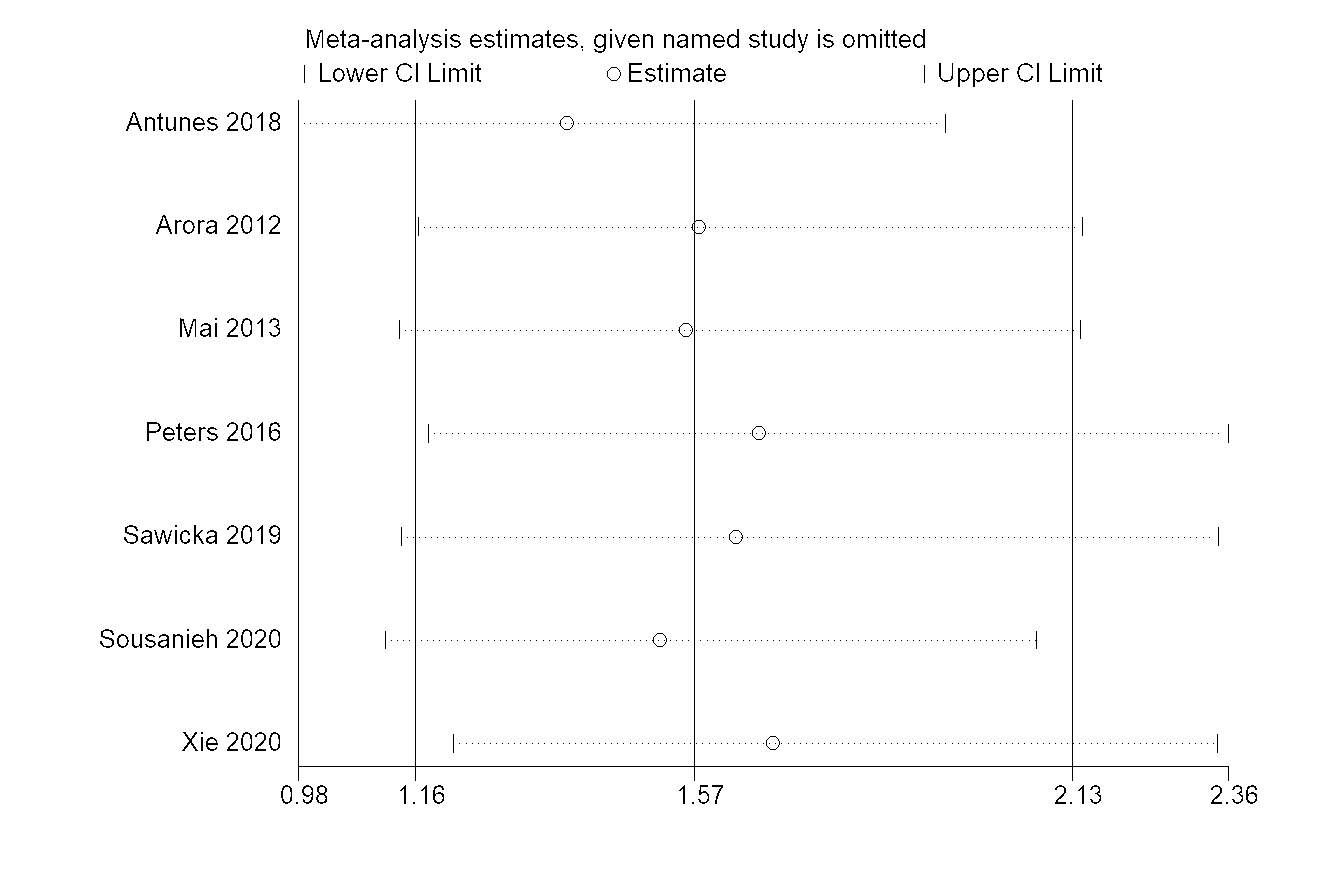

Supplement: Supplemental Material [file IRNF_A_2257806_SM9040.zip › Supplementary Figure 8.tif]
